# Supplementary material for: Resolving the full spectrum of human genome variation using Linked-Reads
Source: Genome Res. 2019 Apr;29(4):635–45. doi: 10.1101/gr.234443.118 (PMC6442396; doi:10.1101/gr.234443.118)
Supplement: Supplemental Material [file supp_gr.234443.118_Supplemental_longranger-2.2.zip › longranger-2.2.tar.bz2!/lr2.2-public/lib/stan/cmdstan/stan/doc/api/html/namespacestan_1_1math_1_1policies_1_1detail.html]

Stan: stan::math::policies::detail Namespace Reference


|  |  |
| --- | --- |
|  | Stan  2.5.0  probability, sampling & optimization |


- Main Page
- Related Pages
- Namespaces
- Classes
- Files

- Namespace List
- Namespace Members

All Classes Namespaces Files Functions Variables Typedefs Enumerations Enumerator Friends Macros Pages

- stan
- math
- policies
- detail

stan::math::policies::detail Namespace Reference

Reimplementing boost::math::policies::detail::raise\_domain\_error.
More...

## Detailed Description

Reimplementing boost::math::policies::detail::raise\_domain\_error.

For error handling, Stan requires raise\_domain\_error to handle two template parameters, one for the return type and the other for the input type.

In boost, these functions are in namespace boost::math::policies:details. Since reimplementing these functions in that namespace could lead to ambiguous functions calls, they are placed in stan::math::policies::details.

These functions still rely on boost::math::policies::detail::raise\_error.

TODO: user\_error may need to be refactored to include the two template parameters.


---

[ Stan Home Page ]

*© 2011–2014,
Stan Development Team.*
